# ABO and Rhesus Blood Groups in Acute Puumala Hantavirus Infection

**DOI:** 10.3390/v13112271

**Published:** 2021-11-13

**Authors:** Johanna Tietäväinen, Outi Laine, Satu Mäkelä, Heini Huhtala, Ilkka Pörsti, Antti Vaheri, Jukka Mustonen

**Affiliations:** 1 Faculty of Medicine and Health Technology, Tampere University, 33520 Tampere, Finland; outi.laine@pshp.fi (O.L.); satu.m.makela@pshp.fi (S.M.); ilkka.porsti@tuni.fi (I.P.); jukka.mustonen@tuni.fi (J.M.); 2 Department of Internal Medicine, Tampere University Hospital, 33520 Tampere, Finland; 3 Faculty of Social Sciences, Tampere University, 33250 Tampere, Finland; heini.huhtala@tuni.fi; 4 Department of Virology, Medicum, University of Helsinki, 00290 Helsinki, Finland; antti.vaheri@helsinki.fi

**Keywords:** Puumala hantavirus, ABO blood groups, rhesus blood groups, capillary leakage, AKI, blood pressure

## Abstract

Puumala hantavirus (PUUV) causes hemorrhagic fever with renal syndrome. We aimed to evaluate whether ABO and rhesus blood groups associate with the susceptibility or the severity of PUUV infection. We analyzed blood groups in 289 adult patients treated in Tampere University hospital due to PUUV infection during the years 1982–2017. Patients’ blood group distribution was compared to that of healthy, voluntary blood donors living in the Tampere University Hospital responsibility area (*n* = 21,833). The severity of PUUV infection, as judged by the severity of acute kidney injury (AKI), thrombocytopenia, inflammation, capillary leakage, and the length of hospital care, was analyzed across the groups. The ABO and rhesus blood group distributions did not differ between the patients and blood donors. Patients with non-O blood groups had lower systolic blood pressure compared to patients with blood group O, but there was no difference in other markers of capillary leakage or in the severity of AKI. Minor deviations in the number of platelets and leukocytes were detected between the O and non-O blood groups. To conclude, patients with blood group O may be less susceptible to hypotension, but otherwise blood groups have no major influences on disease susceptibility or severity during acute PUUV infection.

## 1. Introduction

Puumala hantavirus (PUUV) is a zoonotic pathogen that causes a febrile illness called hemorrhagic fever with renal syndrome (HFRS) in humans. PUUV is a member of *Orthohantavirus* genus in the *Hantaviridae* family, order *Bunyavirales*, and it is the sole human pathogenetic hantavirus in Finland. There are 1000–2500 reported cases annually in Finland with a seroprevalence of 12.5% in the adult population [1,2]. Humans are infected by the vaporized excreta of the bank vole (*Myodes glareolus*) that is the virus’s reservoir. The high season of infection ranges from October to January. The number of infections peaks at 3–4 year intervals tightly following the fluctuation in the number of bank voles [1].

The clinical picture of HFRS includes high fever, acute kidney injury (AKI), thrombocytopenia and increased capillary leakage. Although most cases are mild, the infection-induced capillary leakage can cause a circulatory shock, and about 6% of the hospitalized patients need transient dialysis treatment [1,3]. Bleeding diathesis is rare and case fatality in Finland is less than 0.1% [4]. Host genetic factors influence the clinical course of the disease [5].

The distribution of ABO blood groups differs geographically. A selection pressure caused by infections, the strongest driving force being malaria, has been offered as a possible explanation. In endemic malaria areas, blood group O dominates, and its’ prevalence may reach 90% [6,7,8]. Severe malaria is caused by the intracellular *Plasmodium falciparum* parasite. Infected erythrocytes bind to uninfected erythrocytes forming so called rosettes that may occlude microvascular blood flow and contribute to the development of severe malaria [9]. The rosette formation is reduced in blood group O red cells, thus offering a pathophysiological explanation to the survival benefit provided by blood group O. In dengue fever, severe disease is more common in patients with blood group AB, whereas blood group O is significantly under-represented in the severe hemorrhagic form of dengue fever [10,11,12]. During the 2003 severe acute respiratory syndrome coronavirus (SARS-CoV) outbreak, individuals with blood group O were also less likely to be infected after being exposed to the virus [13], and the same appears to be true in the ongoing SARS-CoV-2 pandemic [14,15,16,17]. In contrast, individuals with blood group A are more prone to SARS-CoV2- infection [14,15,16,17]. However, considerable discrepancy exists whether ABO blood groups affect disease severity. Blood group B individuals have lower risk of hepatitis B (HBV) infection, while blood group O associates with increased risk of norovirus infection [18,19]. Therefore, possible survival advantage provided by different blood groups varies depending on the infective agent and, in many infections, such as in HIV, the relationship with infection and ABO and rhesus blood groups remains controversial or inconclusive [20]. In addition to infections, an association between cancer, cardiovascular diseases, and thrombosis with ABO blood groups, has been detected [21].

Although HFRS caused by PUUV is often a mild disease, PUUV and COVID-19 infections share similar features including AKI, capillary leakage, and a cytokine storm. In the current study we aimed to evaluate whether the susceptibility of PUUV infection, and the severity of the disease, is influenced by ABO and rhesus blood groups.

## 2. Materials and Methods

### 2.1. Subjects

The initial study cohort included 569 adult patients treated in Tampere University Hospital, Finland, due to serologically confirmed acute PUUV infection during the years 1982–2017. The information about ABO and rhesus blood groups were collected retrospectively and was available from 289 (51%) patients. All patients were ethnic Finns (Caucasian).

A detailed medical history of the patients was obtained, and all patients were carefully clinically examined. Blood pressure, heart rate and weight were measured at least daily during hospital care.

Out of the 289 patients, 30 (10%) had one or several previous diagnoses: hypertension (*n* = 12), hyperlipidemia (*n* = 4), type 2 diabetes (*n* = 4), hypothyroidism (4), coronary heart disease (*n* = 3), coeliac disease (*n* = 2), idiopathic thrombocytopenia (2), bronchial asthma (2) and psoriasis (*n* = 2). Two patients were pregnant, and one was breastfeeding. None of the patients had a known chronic kidney disease before the PUUV infection.

The information about the ABO and rhesus blood group distribution in Finland and in the Pirkanmaa district around Tampere University Hospital, Finland, was obtained from the Finnish Red Cross Blood Service. The information included 157,223 first blood donations in Finland, including 21,833 blood donations in Pirkanmaa district, during the years 2000–2010 (Ph.D., Hannele Sareneva, Finnish Red Cross, personal communication), Table 1.

All patients provided a written informed consent, and the study with its’ extensions was approved by the Ethics Committee of the Tampere University Hospital (study codes 96161, 97166, 99256, R04180, R15007 and R09206).

### 2.2. Laboratory Determinations

The diagnosis of PUUV infection was made by detecting the typical granular staining pattern of acute infection and/or low avidity of IgG antibodies in immunofluorescence using PUUV -infected Vero E6 cells as antigens, and/or by detecting PUUV IgM antibodies by an “in-house” enzyme-linked immunosorbent assay based on baculovirus-expressed PUUV nucleocapsid protein. The development and the use of the above and diagnostic methods have been described elsewhere [22].

Plasma creatinine was analyzed by Vitros (Johnson and Johnson, Rochester, NY, USA) until the year 1999 and by Cobas Integra (F. Hoffmann-La Roche Ltd., Basel, Switzerland) from thereafter. Blood cell count was determined by automated hematological cell counters (Bayer Diagnostics, Elkhart, IN, USA) and albumin concentrations using routine automated chemistry analyzers. All laboratory determinations were performed by the Laboratory Centre of the Pirkanmaa Hospital District (later named Fimlab laboratories), Tampere, Finland.

Laboratory analysis from 289 study subjects were available as follows: plasma creatinine 287 (99%), C-reactive protein (CRP) 267 (92%), blood count including hematocrit 276 (96%), leukocytes 278 (96%), platelets 279 (97%), plasma albumin 112 (39%) and ALT in 94/289 (33%). Information about body mass index (BMI) was available from 140 patients (48%). Shock was defined as systolic blood pressure under 90 mmHg and clinical symptoms of a shock such as pale, cold, or clammy skin, rapid breathing, and tachycardia. The information of shock was available from 195 patients (67%). Blood pressure at arrival to the hospital and the minimum blood pressure during hospital care were available from 173 (60%) and 268 (93%) patients, respectively. In the analysis of laboratory variables, minimum or maximum values were used in the statistical analysis as indicated in Table 2, Table 3, Table 4 and Table 5.

### 2.3. Statistical Analysis

The data are presented as medians and ranges for continuous variables and numbers and percentages for categorical variables. Groups were compared using the Kruskal–Wallis test or Mann–Whitney U test, as appropriate. Bonferroni correction for multiple comparisons was applied in the post hoc analyses. The chi-square or Fisher’s exact tests were used to examine differences in proportions, as appropriate. The differences between group means for the initial and minimum blood pressure were calculated with 95% confidence intervals. All analyses were performed using IBM SPSS Statistics version 25 (IBM, Armonk, NY, USA).

## 3. Results

### Clinical and Laboratory

There was no difference in the distribution of ABO or rhesus blood groups between the hospitalized patients with PUUV infection and blood donors in Pirkanmaa district around Tampere University Hospital (*p* = 0.872 for ABO and *p* = 0.660 for rhesus blood groups) Table 1. A similar result was obtained when the blood group distribution of patients was compared with blood donors in Finland (*p* = 0.834 for ABO and *p* = 0.932 for rhesus blood groups).

For disease severity analysis, patients were grouped according to the ABO and rhesus blood groups in three different ways. First, we compared A, B, AB, and O blood groups separately (Table 2 and Table 3). Second, we compared O versus non-O (A, B, and AB combined) blood groups (Table 4 and Table 5). The third grouping comprised a comparison of rhesus positive and negative patients. Different aspects of HFRS including AKI (maximum creatinine), thrombocytopenia (minimum platelet count), inflammation (maximum CRP and leukocyte count) and capillary leakage (weight change, blood pressure upon arrival and minimum blood pressure, signs of clinical shock as well as minimum albumin concentration and maximum hematocrit) were analyzed across the groups.

The clinical characteristics and laboratory findings according to A, B, AB, and O blood groups are presented in Table 2 and Table 3. The initial systolic blood pressure upon arrival to the hospital as well as the minimum blood pressure detected during hospital treatment differed significantly between the groups. In pairwise comparisons with the Bonferroni correction, only blood groups B and O differed significantly for initial and minimum systolic blood pressure. Patients with blood group O had 12.9 mmHg (CI 4.5–21.3 mmHg; *p* = 0.003) higher initial blood pressure and 7.6 mmHg (CI 1.7–13.7 mmHg; *p* = 0.011) higher minimum systolic blood pressure compared to blood group B. 

The effect of ABO blood group on initial blood pressure was similar in both sexes. When grouped according to tertiles or quartiles of age, the effect of blood group O on blood pressure was similar in all age groups (data not shown). Also, when the patients were divided into two groups according to BMI, the effect of blood group on blood pressure remained similar (data not shown).

There were no statistically significant differences in the variables reflecting disease severity namely maximum hematocrit or minimum albumin level (capillary leakage), maximum leukocyte count or CRP (inflammation), minimum platelet count or in maximum plasma creatinine concentration (AKI), between the ABO blood groups, Table 3.

When blood group O was compared with non-O blood groups (Table 4), patients with non-O blood group had lower initial systolic blood pressure and they were more often in clinical shock. The initial systolic blood pressure was 8.6 mmHg (95% CI 2.4–14.7 mmHg; *p* = 0.006) higher in patients with blood group O compared to non-O blood groups.

Patients with blood group O had slightly lower leukocyte count when compared with non-O blood groups, but there was no difference in maximum CRP level (Table 5). Blood group O was associated with lower minimum platelet count.

Altogether 13/289 (4.5%) patients had the diagnosis of hypertension and/or medication affecting blood pressure due to some other illness such as the coronary heart disease. The results remained essentially the same when these patients were excluded from the analysis (data not shown).

In addition, we compared patients expressing antigen A (A and AB combined), B or O, or expressing antigen B (B and AB combined), A or O. The groups again differed significantly in terms of initial and minimum systolic blood pressure. After Bonferroni correction, the only statistically significant differences were detected between blood groups O vs. B and blood groups O vs. B and AB combined. No differences in laboratory parameters were found between the groups.

There were no differences either in the clinical or in the laboratory variables when grouped according to the rhesus factor (data not shown).

## 4. Discussion

We have shown here that ABO and rhesus blood groups do not associate with susceptibility to PUUV infection in Finland. Hospitalized patients with non-O blood groups appear to have lower systolic blood pressure during PUUV infection compared to patients with blood group O. In pairwise analysis, the only significant difference in blood pressure was detected between blood groups O and B. Patients with blood group O have also less severe leukocytosis without a difference in CRP value, but more severe thrombocytopenia when compared with patients with non-O blood groups. To the best of our knowledge, this is the first time the association of blood groups and hantaviral infections has been investigated.

ABO blood groups have a role beyond transfusion medicine and are considered a part of the innate immune system [21]. The ABO histo-blood groups consist of A and B antigens that are co-dominant products of a single ABO gene, and an H antigen, that is a result of homozygous inheritance of two null ABO alleles. The system creates four blood types (A, B, AB and O) [23]. Group O individuals express the H antigen, which is the biosynthetic precursor of the A and B antigens. In addition to red cells, ABH antigens are expressed in the endothelium, platelets, intestinal mucosa, kidney, heart, gastrointestinal tract, female reproductive organs, as well as in fluids and secretions including saliva, mucus, and plasma. However, the level of ABH expression both in cells and in secretions between individuals of the same blood type varies greatly, depending on the other histo-blood group antigens, namely secretor status and the expression of Lewis antigen [23].

The expression of ABH antigens in different oligosaccharide backbones influences the recognition of antigens by antibodies and by microbes. As microbes often use glycosylated cell-surface receptors for their attachment and entry into cells, it is conceivable that ABH antigens may play a role in determining susceptibility to infections [21,23]. Accordingly, histo-blood group antigens function as a receptor or attachment factors on gut epithelial surfaces. Children with blood groups A and AB are more prone to rotavirus gastroenteritis compared to children with blood group O. The resistance of infection is also dependent on the secretor and Lewis antigen status as well as the rotavirus genotype, which varies geographically [24].

In the current study, no differences in the susceptibility of PUUV infection in relation to either ABO or rhesus blood groups were detected. As the study participants consist solely of hospitalized patients with a relatively severe disease, the detection of a clinically significant association is possible even in a relatively small sample size. Accordingly, other genetic factors such as the HLA B8-DR3 phenotype, have been shown to be more prevalent and to associate with a more severe disease in hospitalized patients with PUUV infection [25].

In SARS-CoV-2 infection a relation between ABO blood groups and infection susceptibility has been found with lower susceptibility in subjects with blood group O. The body produces natural antibodies against those ABO blood group antigens that are not expressed in the own cells [23]. In SARS-CoV-2, the titer and types of natural antibodies (IgM vs. IgG), spatial hindrance of viral attachment to its receptor by the bound antibodies, and destruction of the antibody coated viruses by the complement, have been presented as explanations for the lower incidence of infection in blood group O individuals [26,27,28,29,30]. Moreover, rhesus-positive individuals have been suggested to be more prone to severe SARS-CoV-2 disease [31].

In the present study, patients with non-O blood groups, especially those with blood group B, had lower systolic blood pressure compared to blood group O. In two recent studies an association with blood group O and hypertension was observed [32,33]. In our study, only 12/289 (4%) patients had a diagnosis of hypertension before hospital treatment and there was no statistically significant difference in hypertension prevalence between blood groups (data not shown). Whether the differences in blood pressure detected during PUUV infection represent a true susceptibility to hypotension in patients with non-O blood groups or is more related to the higher likelihood of hypertension in blood group O patients needs further evaluation. As other markers of capillary leakage, namely the level of maximum hematocrit, minimum plasma albumin concentration, or change of weight during hospital treatment, did not differ in the blood groups, increased capillary leakage is not a likely explanation for the observed difference in blood pressure.

No clinically significant difference in blood cell counts in relation to ABO blood groups has been detected in large population-based studies [32]. However, during PUUV infection, minimum platelet count and maximum leukocyte count were lower in patients with blood group O when compared with the other blood groups. The reason for this is currently unknown.

Platelet activation and thrombus formation result from the complex interplay between the vascular endothelium, plasma proteins and platelets. Increased platelet consumption on the injured endothelium is considered to be the mechanism of thrombocytopenia in hantavirus infections, and the level of several markers of platelet activation and aggregation are altered during PUUV infection [1,34]. Fibrinogen and von Willebrand factor (VWF) are upregulated in the acute phase, whereas the levels of a disintegrin and metalloproteinase with thrombospondin type 1 domain 13 (ADAMTS13) activity and fibronectin are downregulated. Fibrinogen level also negatively correlates with thrombocytopenia [35]. The function of VWF is to mediate platelet activation and aggregation to the injured endothelium. It is currently not known whether the results above differ in relation to blood groups during infection. However, in a steady state, 20–25% lower level of VWF and factor VIII are detected in the serum of blood group O individuals [23,36]. In contrast, blood group O carriers exhibit higher concentrations of endothelium-derived soluble adhesion molecules (P-selectin, E-selectin and soluble intercellular adhesion molecule-1 (sICAM-1)), while ABH antigenicity is even expressed on the platelet endothelial cell adhesion molecule-1 (PECAM-1) [36]. The circulating level of soluble P-selectin, as well as soluble glycoprotein VI, are also influenced by acute PUUV infection [37].

ABO glycosyltransferases modify platelet surface glycoproteins (GP), glycolipids and glycosphingolipids, many of which participate in platelet activation and thrombosis via different mechanisms [21,23,36]. It is possible that ABO modifications result in changes of platelet function. Of these, GP IIb/IIIa complex mediates platelet activation and aggregation via the binding of fibrinogen, fibronectin and VWF. Interestingly, GP IIIA—also named β3 integrin—is a receptor for hantaviruses. In addition to platelets, it is expressed on endothelial cells and macrophages. Hantaviruses bind quiescent platelets preventing their activation and direct them to adhere to the endothelial cells via β3 integrins [34]. Accordingly, platelets from PUUV-infected patients show impaired aggregation to stimuli other than collagen [38].

In PUUV infection, neutrophils are activated by PUUV-infected endothelial cells, and the activation is more likely to occur indirectly via virus-infected microvascular endothelial cells rather than directly through virus contacts with neutrophils [39]. Neutrophil activation leads to the release of neutrophil extracellular traps (NETs) and/or release of antimicrobial proteins (degranulation). As an indication of NETosis in PUUV infection, histones and cell free DNA (cfDNA) are detected in plasma [39,40,41]. In addition, it is currently appreciated that platelets are crucial in neutrophil activation and NET production [42]. Therefore, platelet and leukocyte number and function are interrelated with ABO blood groups in a multitude of ways, which may explain the observed differences in the number of platelets and leukocytes in this study.

The association of ABO blood groups with different diseases has a been a subject of investigation and debate for decades. It appears that a true connection is evident but the pathophysiological mechanisms as well as the extent of the effects in different diseases vary. Although the investigations concerning ABO blood groups have dominated, a connection between secretor status, Lewis antigen as well as rhesus (Rh) blood group with diseases has also been made [23].

## 5. Conclusions

According to our results, ABO and rhesus blood groups do not influence the susceptibility to PUUV infection. Patients with non-O blood group may be more prone to hypotension and the number of platelets and leukocytes during PUUV infection may be affected by ABO blood groups.

## Figures and Tables

**Table 1:** ABO and rhesus blood group distribution in patients with Puumala hantavirus (PUUV) infection and in blood donors in the Pirkanmaa district, Finland.

	Patients *n* = 289		Blood Donors in Pirkanmaa *n* = 21,833	
	Number	%	Number	%
A	126	43.6	8989	41.2
B	48	16.6	3733	17.1
AB	22	7.6	1741	8.0
O	93	32.2	7370	33.8
Rh+	249	86.2	19002	87.0
Rh−	40	13.8	2831	13.0

**Table 2:** Clinical findings in 289 patients with PUUV infection according to ABO blood group.

	A *n* = 126		B *n* = 48		AB *n* = 22		O *n* = 93		*p* Value
	Median/Number	Range/%	Median/Number	Range/%	Median/Number	Range/%	Median/Number	Range/%	
Age (years)	38.9	20.4–68.9	35.3	19.7–61.2	35.4	21.0–58.2	41.4	21.5–65.7	0.257
Male/female	89/37	71/29	35/13	73/27	12/10	55/45	66/27	71/29	0.440 ^†^
BMI	24.7	17.5–35.4	22.0	18.9–35.8	25.3	18.6–37.6	24.7	20.6–44.2	0.092
Shock ^τ^	7/87	8.0	3/35	8.6	0/15	0	0/58	0	0.078 *
Systolic BP initial (mmHg)	125	60–180	120	90–158	131	105–168	135	100–174	0.010
Diastolic BP initial (mmHg)	80	30–110	75	0–95	85	60–100	80	60–112	0.167
Min systolic BP (mmHg)	120	60–160	110	80–180	120	90–160	120	74–155	0.036
Min diastolic BP (mmHg)	72	36–100	70	40–109	75	60–90	70	50–90	0.482
Weight change (kg) ^~^	3.2	0.2–18.5	2.9	0.4–12.9	2.0	0.5–11.6	3.0	0.3–18.5	0.710
Dialysis	8/87	9.2	5/35	14.3	0/15	0	8/59	13.6	0.423 *
Hospital stay (days)	8	1–30	9	2–46	7.5	4–66	8	2–27	0.589

^τ^ Shock defined as systolic blood pressure under 90 mmHg and clinical symptoms of a shock. BMI, body mass index; BP, blood pressure; ^~^ Difference between highest and lowest weight during hospital care reflecting both capillary leakage and fluid accumulation during oliguric phase. Fisher’s exact test is marked with an asterisk (*), chi-square with (^†^), all others are Kruskal–Wallis H-tests.

**Table 3:** Laboratory findings in 289 patients with PUUV infection according to ABO blood group.

	A *n* = 126		B *n* = 48		AB *n* = 22		O *n* = 93		*p* Value *
Plasma and Blood Findings	Median	Range	Median	Range	Median	Range	Median	Range	
Hematocrit max^~^	0.43	0.34–0.66	0.42	0.32–0.64	0.41	0.35–0.56	0.42	0.26–0.62	0.671
Albumin min (g/L) ^τ^	29	11–45	30	22–39	28	24–34	29	19–43	0.756
Leukocytes max (x10^9^/L) ^~^	10.4	4.5–39.1	9.7	4.4–50.3	8.9	4.9–19.8	8.9	3.8–44.9	0.092
CRP max (mg/L)^~^	70	11–200	69	13–156	72	11–280	67	12–214	0.843
Platelets min (x10^9^/L) ^τ^	73	4–378	66	3–264	83	18–311	60	9–332	0.166
Creatinine max (µmol/L) ^~^	265	71–1645	317	67–1537	309	71–1183	200	70–1290	0.371
ALT max U/L	44	8–2076	51	20–1892	29	10–102	41	16–109	0.587

* Kruskal–Wallis H-tests; ^~^ maximum value during hospitalization; ^τ^ minimum value during hospitalization; CRP, C-reactive protein; ALT, alanine aminotransferase.

**Table 4:** Clinical findings in 289 patients with PUUV infection in non-O (A, B and AB) blood group against O blood group.

	Non-O (A, B, AB) *n* = 196		O *n* = 93		*p* Value
	Median/Number	Range/%	Median/Number	Range/%	
Age (years)	38.6	19.7–68.9	41.4	21.5–65.7	0.088
Male/female	136/60	69/31	66/27	71/29	0.891 ^†^
BMI	24.3	17.5–37.6	24.7	20.6–44.24	0.218
Shock ^τ^	10/137	7.3	0/58	0	0.035 *
Systolic BP initial (mmHg)	125	60–180	135	100–174	0.006
Diastolic BP initial (mmHg)	80	30–110	80	60–112	0.173
Min systolic BP (mmHg)	119	60–180	120	74–155	0.063
Min diastolic BP (mmHg)	70	36–109	70	50–90	0.786
Weight change (kg) ^~^	3	0.2–18.5	3	0.3–18.5	0.550
Dialysis	13/137	9.5	8/59	13.6	0.452 ^†^
Hospital stay (days)	8	1–66	8	2–27	0.287

^τ^ Shock defined as systolic blood pressure under 90 mmHg and clinical symptoms of a shock. BMI, body mass index; BP, blood pressure; ^~^ Difference between highest and lowest weight during hospital care reflecting both capillary leakage and fluid accumulation during oliguric phase. Fisher’s exact test is marked with an asterisk (*), chi-square with (^†^), all others are Kruskal–Wallis H-tests.

**Table 5:** Laboratory findings in 289 patients with PUUV infection in non-O (A, B and AB) blood group against O blood group.

	Non-O (A, B, AB) *n* = 196		O *n* = 93		*p* Value *
Plasma and Blood Findings	Median	Range	Median	Range	
Hematocrit max ^~^	0.42	0.32–0.66	0.42	0.26–0.62	0.830
Albumin min (g/L) ^τ^	29	11–45	29	19–43	0.726
Leukocytes max (x10^9^/L) ^~^	10.1	4.4–50.3	8.9	3.8–44.7	0.047
CRP max (mg/L) ^~^	70	11–280	67	12–214	0.459
Platelets min (x10^9^/L) ^τ^	71	3–378	60	9–332	0.041
Creatinine max (µmol/L) ^~^	278	67–1645	200	70–1290	0.086
ALT max U/L	44	8–2076	41	16–109	0.832

* Kruskal–Wallis H-tests; ^~^ maximum value during hospitalization; ^τ^ minimum value during hospitalization; CRP, C-reactive protein; ALT, alanine aminotransferase.

## Data Availability

Original data are available as Supplementary Materials.

## Supplementary Materials

The following are available online at https://www.mdpi.com/article/10.3390/v13112271/s1, The original data are available as Supplementary Materials.

## References

[1] Vaheri A., Strandin T., Hepojoki J., Sironen T., Henttonen H., Mäkelä S., Mustonen J. (2013). Uncovering the Mysteries of Hantavirus Infections. Nat. Rev. Microbiol..

[2] Latronico F., Mäki S., Rissanen H., Ollgren J., Lyytikäinen O., Vapalahti O., Sane J. (2018). Population-Based Seroprevalence of Puumala Hantavirus in Finland: Smoking as a Risk Factor. Epidemiol. Infect..

[3] Mustonen J., Outinen T., Laine O., Pörsti I., Vaheri A., Mäkelä S. (2017). Kidney Disease in Puumala Hantavirus Infection. Infect. Dis..

[4] Mustonen J., Mäkelä S., Outinen T., Laine O., Jylhävä J., Arstila P.T., Hurme M., Vaheri A. (2013). The Pathogenesis of Nephropathia Epidemica: New Knowledge and Unanswered Questions. Antiviral Res..

[5] Mustonen J., Partanen J., Kanerva M., Pietilä K., Vapalahti O., Pasternack A., Vaheri A. (1996). Genetic Susceptibility to Severe Course of Nephropathia Epidemica Caused by Puumala Hantavirus. Kidney Int..

[6] Degarege A., Gebrezgi M.T., Ibanez G., Wahlgren M., Madhivanan P. (2019). Effect of the ABO Blood Group on Susceptibility to Severe Malaria: A Systematic Review and Meta-Analysis. Blood Rev..

[7] Panda A.K., Panda S.K., Sahu A.N., Tripathy R., Ravindran B., Das B.K. (2011). Association of ABO Blood Group with Severe Falciparum Malaria in Adults: Case Control Study and Meta-Analysis. Malaria J..

[8] Cserti C.M., Dzik W.H. (2007). The ABO Blood Group System and Plasmodium Falciparum Malaria. Blood.

[9] Rowe A.J., Handel I.G., Mahamadou T.A., Deans A., Lyke K.E., Koné A., Diallo D.A., Raza A., Kai O., Marsh K. (2007). Blood Group O Protects Against Severe Plasmodium Falciparum Malaria through the Mechanism of Reduced Rosetting. Proc. Natl. Acad. Sci. USA.

[10] Murugananthan K., Subramaniyam S., Kumanan T., Owens L., Ketheesan N., Noordeen F. (2018). Blood Group AB is Associated with Severe Forms of Dengue Virus Infection. Virus Dis..

[11] Ravichandran S., Ramya S.R., Kanungo R. (2019). Association of ABO Blood Groups with Dengue Fever and its Complications in a Tertiary Care Hospital. J. Lab Physicians.

[12] Kalayanarooj S., Gibbons R.V., Vaughn D., Green S., Nisalak A., Jarman R.G., Mammen M.P., Perng G. (2007). Blood Group AB is Associated with Increased Risk for Severe Dengue Disease in Secondary Infections. J. Infect. Dis..

[13] Cheng Y., Cheng Y., Cheng G., Chui C.H., Lau F.Y., Chan P.K.S., Ng M.H.L., Sung J.J.Y., Wong R.S.M. (2005). ABO Blood Group and Susceptibility to Severe Acute Respiratory Syndrome. JAMA.

[14] Golinelli D., Boetto E., Maietti E., Maria P.F. (2020). The Association between ABO Blood Group and SARS-CoV-2 Infection: A Meta-Analysis. PLoS ONE.

[15] Leaf R.K., Al-Samkari H., Brenner S.K., Gupta S., Leaf D.E. (2020). ABO Phenotype and Death in Critically Ill Patients with COVID-19. Br. J. Haematol..

[16] Liu N., Zhang T., Ma L., Zhang H., Wang H., Wei W., Pei H., Li H. (2020). The Impact of ABO Blood Group on COVID-19 Infection Risk and Mortality: A Systematic Review and Meta-Analysis. Blood Rev..

[17] Wu B., Gu D., Yu J., Yang J., Shen W. (2020). Association between ABO Blood Groups and COVID-19 Infection, Severity and Demise: A Systematic Review and Meta-Analysis. Infect. Genet Evol..

[18] Jing W., Zhao S., Liu J., Liu M. (2020). ABO Blood Groups and Hepatitis B Virus Infection: A Systematic Review and Meta-Analysis. BMJ Open.

[19] Liao Y., Xue L., Gao J., Wu A., Kou X. (2020). ABO Blood Group-Associated Susceptibility to Norovirus Infection: A Systematic Review and Meta-Analysis. Infect. Genet Evol..

[20] Davison G.M., Hendrickse H.L., Matsha T.E. (2020). Do Blood Group Antigens and the Red Cell Membrane Influence Human Immunodeficiency Virus Infection?. Cells.

[21] Franchini M., Bonfanti C. (2015). Evolutionary Aspects of ABO Blood Group in Humans. Clin. Chim. Acta.

[22] Vaheri A., Vapalahti O., Plyusnin A. (2008). How to Diagnose Hantavirus Infections and Detect them in Rodents and Insectivores. Rev. Med. Virol..

[23] Cooling L. (2015). Blood Groups in Infection and Host Susceptibility. Clin. Microbiol. Rev..

[24] Pérez-Ortín R., Vila-Vicent S., Carmona-Vicente N., Santiso-Bellón C., Rodríguez-Díaz J., Buesa J. (2019). Histo-blood group antigens in children with symptomatic rotavirus infection. Viruses.

[25] Mäkelä S., Mustonen J., Ala-Houhala I., Hurme M., Partanen J., Vapalahti O., Vaheri A., Pasternack A. (2002). Human Leukocyte Antigen-B8-DR3 is a More Important Risk Factor for Severe Puumala Hantavirus Infection than the Tumor Necrosis Factor-A(-308) G/A Polymorphism. J. Infect Dis..

[26] Focosi D. (2020). Anti-A Isohaemagglutinin Titres and SARS-CoV-2 Neutralization: Implications for Children and Convalescent Plasma Selection. Br. J. Haematol..

[27] Gérard C., Maggipinto G., Minon J. (2020). COVID-19 and ABO Blood Group: Another Viewpoint. Br. J. Haematol..

[28] Li J., Wang X., Chen J., Cai Y., Deng A., Yang M. (2020). Association between ABO Blood Groups and Risk of SARS-CoV-2 Pneumonia. Br. J. Haematol..

[29] Deleers M., Breiman A., Daubie V., Maggetto C., Barreau I., Besse T., Clémenceau B., Ruvoën-Clouet N., Fils J., Maillart E. (2021). Covid-19 and Blood Groups: ABO Antibody Levels may also Matter. Int. J. Infect. Dis..

[30] Zaidi F.Z., Zaidi A.R.Z., Abdullah S.M., Zaidi S.Z.A. (2020). COVID-19 and the ABO Blood Group Connection. Transfus. Apher. Sci..

[31] Taha S.A.H., Osman M.E.M., Abdoelkarim E.A.A., Holie M.A.I., Elbasheir M.M., Abuzeid N.M.K., Al-Thobaiti S., Fadul S.B., Konozy E.H.E. (2020). Individuals with a Rh-Positive but Not Rh-Negative Blood Group are More Vulnerable to SARS-CoV-2 Infection: Demographics and Trend Study on COVID-19 Cases in Sudan. New Microbes New Infect..

[32] Groot H.E., Villegas Sierra L.E., Said M.A., Lipsic E., Karper J.C., van der Harst P. (2020). Genetically Determined ABO Blood Group and its Associations with Health and Disease. Arterioscler. Thromb. Vasc. Biol..

[33] Li S., Schooling C.M. (2020). A Phenome-Wide Association Study of ABO Blood Groups. BMC Med..

[34] Gavrilovskaya I.N., Gorbunova E.E., Mackow E.R. (2010). Pathogenic Hantaviruses Direct the Adherence of Quiescent Platelets to Infected Endothelial Cells. J. Virol..

[35] Laine O., Mäkelä S., Mustonen J., Helminen M., Vaheri A., Lassila R., Joutsi-Korhonen L. (2011). Platelet Ligands and ADAMTS13 during Puumala Hantavirus Infection and Associated Thrombocytopenia. Blood Coagul. Fibrinol..

[36] Zhong M., Zhang H., Reilly J.P., Chrisitie J.D., Ishihara M., Kumagai T., Azadi P., Reilly M.P. (2015). ABO Blood Group as a Model for Platelet Glycan Modification in Arterial Thrombosis. Arterioscler. Thromb. Vasc. Biol..

[37] Connolly-Andersen A., Sundberg E., Ahlm C., Hultdin J., Baudin M., Larsson J., Dunne E., Kenny D., Lindahl T.L., Ramström S. (2015). Increased Thrombopoiesis and Platelet Activation in Hantavirus-Infected Patients. J. Infect. Dis..

[38] Laine O., Joutsi-Korhonen L., Lassila R., Koski T., Huhtala H., Vaheri A., Mäkelä S., Mustonen J. (2015). Hantavirus Infection-Induced Thrombocytopenia Triggers Increased Production but Associates with Impaired Aggregation of Platelets Except for Collagen. Thromb. Res..

[39] Strandin T., Mäkelä S., Mustonen J., Vaheri A. (2018). Neutrophil Activation in Acute Hemorrhagic Fever with Renal Syndrome is Mediated by Hantavirus-Infected Microvascular Endothelial Cells. Front. Immunol..

[40] Outinen T.K., Kuparinen T., Jylhävä J., Leppänen S., Mustonen J., Mäkelä S., Pörsti I., Syrjänen J., Vaheri A., Hurme M. (2012). Plasma Cell-Free DNA Levels are Elevated in Acute Puumala Hantavirus Infection. PLoS ONE.

[41] Raftery M.J., Lalwani P., Krautkrӓmer E., Peters T., Scharffetter-Kochanek K., Krüger R., Hofmann J.ö., Seeger K., Krüger D.H., Schönrich G. (2014). Β2 Integrin Mediates Hantavirus-Induced Release of Neutrophil Extracellular Traps. J. Exp. Med..

[42] Kim S., Jenne C.N. (2016). Role of Platelets in Neutrophil Extracellular Trap (NET) Production and Tissue Injury. Semin. Immunol..

