# Supplementary material for: ABO and Rhesus Blood Groups in Acute Puumala Hantavirus Infection
Source: Viruses. 2021 Nov 13;13(11):2271. doi: 10.3390/v13112271 (PMC8621274; doi:10.3390/v13112271)
Supplement: Supplementary file 1 [file viruses-13-02271-s001.zip › viruses-1423706-supplementary.pdf]

Age	Sex (male 0, femal	Hospital stay ((	Syst BP initia	Dia BP initia	Weight cha	BMI	Dialysis	Shock	Syst BP ma	Syst BP mi	Dia BP ma	Dia BP min
58,18	0	15										
40,13	0	10	130	80	1,1				130	122	90	80
25,45	0	66			11,6				170	111	100	70
48,64	1	9	125	85	5,4	27,4	0	0	178	125	105	85
45,42	1	7	130	85	2	37,55	0	0	130	120	85	80
43,35	1	5	140	80	1,2	25,35	0	0	140	108	80	68
40,69	0	9	140	60	4,5	22,18	0	0	138	134	89	79
30,35	0	6			2				130	130	90	90
31,64	0	9			0,9				106	106	66	66
30,89	0	17			6,9				160	120	100	70
33,14	0	8	130	100	6,9	28,5	0	0	130	116	100	77
56,12	1	4	168	88	0,5	27,01	0	0	168	120	88	70
30,02	1	6	105	95	0,7	21,41	0	0	120	105	85	72
26,7	1	7			2,1		0	0	95	90	60	60
20,99	0	5	132	96	1,6	18,57	0	0	132	130	96	80
24,2	0	7	105	75	1,9		0	0	105	105	75	75
22,45	1	7	110	70	1	19,88	0	0	110	110	70	70
37,58	1	4			0		0	0	127	103	81	71
24,85	0	5	132	78	3	25,62	0	0	132	132	78	78
54,73	1	12	160	90	4,6	25,19	0	0	180	160	90	78
48,32	0	8	140	85			0	0				
50,78	1	12										
48,39	1	8			0				105	105	80	80
21,54	0	7			4,8				140	138	85	72
24,8	0	8			5,8				140	135	85	80
41,43	0	8	119	82	4,8	27,52	0	0	155	119	95	80
37,21	0	8	130	75	0		0	0	130	130	75	75
65,69	0	9	170	85	4,5	23,88	0	0	170	130	90	80
31,96	1	7	105	80	1,6	31,56	0	0	128	105	80	68
52,06	0	8	135	85	1,6	26,75	1	0	150	124	85	65
45,59	0	19	150	95	12,8		1	0	175	125	90	50
30,58	0	8										

43,51	0	5	160	90	2,4	27,44	0	0	171	155	102	85
40,82	0	9	140	75	8	24,65	1	0	150	135	79	72
50,81	0	5	145	95	0	25,16	0	0	142	131	95	87
61,88	0	13	152	86	3,2	23,09	1	0	186	114	104	70
43,26	0	2			0,3				138	129	70	67
59,2	1	5										
55,51	0	7			18,5				160	109	84	65
29,68	1	10			8,4				165	120	90	70
27	0	9			5,7				152	120	95	80
57,94	1	19			5,2				150	140	90	70
31,65	0	4			0				105	105	60	60
56,18	1	6			0,6				140	118	78	70
21,74	0	5			0				130	124	84	80
43,69	0	8			3,4				130	130	80	50
25,7	0	4			1,7				142	142	80	80
39,03	0	9			2,6				116	100	80	64
36,61	0	7										
56,06	0	9			4,2				125	95	75	50
40,07	1	8			0,7				125	125	70	70
27,76	0	6			0,7				120	110	75	55
61,4	1	16			0				130	130	70	70
51,64	0	11			2				120	100	75	60
23,62	1	5	130	80	1	22,31			130	130	80	80
36,4	0	11			7,2				120	110	80	80
38,29	0	11			5,3				120	115	100	80
32,52	0	7			3,5				140	138	85	78
39,42	0	8			0,9				145	134	90	80
33,43	0	10			2,1				154	130	100	85
44,68	0	13			4,9				175	130	118	90
47,18	0	5	140	95	1	25,47	0	0	140	120	95	80
41,09	0	14	150	80	8,7		0	0	160	134	85	60
49,66	1	10	150	95	4,6		0	0	150	120	95	70
36,88	0	9			10,2		0	0	160	128	100	70

23,23	0	7	144	98	1,2		0	0	144	118	104	80
48,95	0	8	150	95	0,4		0	0	160	132	80	60
36,79	0	5	100	80	3	24,61	0		112	90	82	60
56,49	0	7	135	95			0	0				
43,49	0	4	110	70	1,1	29,8	0	0	120	110	78	60
29,12	1	13					0	0	110	95	75	70
56,9	1	6	130	75	1	21,33	0	0	130	110	75	60
32,33	1	8	120	80	3,7	29,61	0	0	130	120	85	80
42,42	0	8	140	85	0,7		0	0	140	116	85	78
35,72	0	11	140	70	1,4		0	0	140	100	80	64
47,92	0	7	140	100	3	30,42	0	0	154	135	102	88
36,33	1	8	105	65	3,7	22,7	0	0	110	90	72	60
47,14	1	9	130	70	3,8	35,1	0	0	160	120	80	70
45,17	0	6	150	100	0,8		0	0	150	140	100	90
35,08	0	5	135	80	0,4	22,67	0	0	135	130	80	75
46,15	1	8	110	80	2,9	26,32	0	0	140	110	90	80
33,4	0	11	130	60	3,2	24,91	0	0	130	110	80	60
38,66	0	7	140	85	0,7	24,76	0	0	180	135	85	60
31,79	0	9	150	70	0,4	28,38	0	0	155	150	80	70
30,48	0	11	160	100	2,9	24,27	0	0	160	135	100	75
44,01	0	7	150	90	3,1	20,63	0	0	150	150	90	70
36,24	0	7	100	60	1,1	22,92	0	0	106	100	75	68
43,42	0	12			5,5		0	0	125	115	80	70
43,42	0	12			4,5	20,95	0	0	132	130	90	80
25,36	0	6			1,3		0	0	120	120	80	80
55,18	1	12			2,4		0	0	155	74	85	65
33,67	0	8	130	80	0,9		0	0	130	114	80	65
42,77	1	7	174	112	3,7	22,72	0	0	174	130	112	70
53,07	1	8	100	60	2,8	23,31	0	0	132	100	94	60
50,4	1	27	100	60	16,1	44,24	1	0	210	100	110	70
47,19	0	14	100	70	4,7	22,04	1	0	120	100	80	60
40,19	0	8	120	78	6,4		1	0	162	120	98	78
63,69	0	7	130	74	5,7	23,62	0	0	161	130	93	74

39,6	0	10			2,1				145	110	90	64
52,39	0	7			3,3				146	126	92	77
26,69	1	9										
33,43	1	4	114	80	1,3	22,21	0	0	114	100	80	64
44,05	1	10	120	80	6,4	30,3	0	0	140	116	90	80
44,58	1	5	140	92	0,8	26,37	0	0	140	125	92	70
64,14	1	5	160	95	0,5		0	0	160	120	95	70
36,8	0	9	110	75	1,5	23,47	0	0	110	110	75	75
46,01	0	7	115	78	3,8	28,87	0	0	144	115	99	78
29,4	0	8	134	80	2,1	21,11	0	0	134	126	80	66
24,11	0	5	155	75	2,1	26,07	0	0	165	155	104	75
42,4	1	6	130	90	1,8	31,72	0	0	160	115	86	77
60,36	1	7			0,7				150	132	80	70
33,51	0	6			3,1				148	128	90	76
47,47	0	17	135	90	8,7	25,12	0	0	180	126	120	80
56,85	0	10	145	60	7,3	21,87	0	0	150	116	109	60
37,68	0	6	145	110	6,2	22,45	1	0	145	140	110	80
44,03	0	7			0,5				140	110	85	60
28,58	0	11			5,3				120	110	70	50
29,34	0	18	110	65	2,1		0	0	112	100	78	60
24,87	0	15	100	60	10,2	24,1	0	0	150	100	95	60
54,67	1	12	138	70	1,6		0	0	138	114	74	70
33,61	1	12	110	60	2,8	19,96	1	0	147	106	97	72
51,12	0	6	125	84	4,1	29,53	0	0	125	120	100	80
56,94	0	5	120	84	1,5	35,75	0	0	136	120	92	84
47,55	1	8	90		3,3	31,44	0	1	166	90	105	87
27,81	1	10			4,7				162	100	65	58
44,79	1	5			1,2				120	120	74	74
52,61	0	9			2,8				170	144	100	80
46,55	1	18			12,9				180	120	100	80
58,95	0	8			1,4				160	145	100	85
33,57	0	9	145	95	6,2						95	
19,72	0	12			5,9				180	180	80	80

25,7	0	11			5,1				160	138	96	82
21,62	1	6	130	60	0,4	20,23	0	0	130	130	60	60
60,06	0	10	104	68	4,7	26,46	0	0	156	104	92	67
49,29	0	4	130	85	3	29,94	0	0	130	130	85	85
29,45	1	3	120	85	0,4		0	0	122	110	85	68
46,84	0	18	150	85	11,8	24,98	0	0	180	115	102	64
29,99	0	6			1,4		0	0	136	110	90	80
21,89	0	6	118	88	6,3	25,7	0	0	140	90	82	50
32,87	0	8	120	70	1,3	22,68	0	0	120	104	75	70
40,43	0	9			0,4	20,77	0	0	124	124	84	84
39,6	0	10			2,7	21,85	0	0	134	110	85	58
29,53	1	8	125	90	0,4	19,48	0	0	125	125	90	90
31,55	0	7			2	30,93	0	0	130	120	90	70
25,42	0	9	145	85	1,4	21,05	0	0	145	140	85	80
51,79	0	10	125	85	4,8		0	0	160	120	85	70
40,25	0	7			2,9		0	0	130	120	90	70
23,16	1	7	135	85	2,2	18,93	0	0	135	110	85	65
29,59	0	6	158	78	0	20,76	0	0	156	110	90	60
31,42	0	9	105	55	4,7		0	0	155	120	105	78
56,21	0	5			0,9	24,22	0	0	174	92	174	109
30,05	0	9	120	75	4,9	18,92	0	0	132	110	81	69
36,97	0	20	105	90	6,2	20,9	1	0	160	105	90	70
52,59	0	46					1	1				
33,63	0	2			0				120	120	80	70
51,67	1	11	116	90	5,8	23,15	0	0	180	105	108	70
25,56	1	5	105	70	2,5	21,09	0	0	116	105	96	68
32,69	0	6	135	75	3,7	24,39	0	0	135	109	75	72
28,21	0	5	115	60	1,4	21,68	0	0	113	93	71	61
57,76	1	11	104	72	10,1	22,04	1	0	129	96	76	51
39,12	0	8	90	70	1,7	20,38	1	1	123	90	79	60
61,24	0	21			8				180	90	80	60
45,7	0	15							145	80	95	40
30,35	0	8			3,2				170	134	85	75

38,37	0	8			0				140	140	75	75
29,37	0	8			1,3				145	125	90	75
68,86	1	8	110	80			0	0				
51,74	0	5			0,9	23,56	0	0	115	110	65	60
31,64	0	6	140	90	2	22,5	0	0	140	124	90	76
50,17	0	9	155	80	1,8	25,82	0	0	155	130	82	80
49,1	0	7	140	80	2,4	22,68	0	0	140	140	80	80
45,12	0	10	126	84	4		0	0	140	120	95	70
27,56	1	12			5,7		0	0	115	100	80	55
30,74	0	5	125	70	6,9		0	0	125	120	70	60
33,58	0	9	150	85	4,3	25,92	0	0	150	150	85	85
53,59	0	6	130	75	0,5	26,76	0	0	130	118	76	67
54,69	0	17			18,5		0	1	150	70	80	40
44,48	1	10	124	88			0	0	160	124	92	80
46,4	0	7	110	90	1,4	29,98	0	0	140	100	100	70
61,73	0	14			3,1	26,44	0	0	160	150	100	80
41,83	1	8	140	80	4	27,37	0	0	160	140	95	80
56,25	1	5	142	84	1,6	28,8	0	0	142	114	84	75
49,74	0	19	108	70	11,2	28,77	1	0	150	98	90	75
29,16	1	7	120	78	2,2	23,04	0	0	184	116	86	74
44,24	0	7	125	75	1,3		0	0	132	120	90	60
38,77	0	4			0,9	35,43			140	105	82	50
26,43	0	5			1,1				140	140	80	80
31,96	1	5							105	105	80	80
53,81	0	7			2,7				130	104	80	64
33,23	1	5			1,4				112	95	68	60
29,18	0	3			0,9				140	136	85	80
41,64	0	2	140	90					140	140	90	90
26,46	1	12										
44,59	1	8			1,8				120	85	80	40
50,56	0	8			2,2				155	130	90	80
28,7	0	6			0,9				110	110	70	70
40,09	0	10			5,8				150	150	85	85

44,04	0	12			5				125	98	85	60
55,85	1	10			4				160	160	90	90
42,22	0	11			5,5				165	125	100	80
43,83	0	7	155	100	0							
41,18	1	14			5,1				100	100	55	55
27,3	0	12			10,8				170	145	115	90
38,34	0	12			4,6				145	132	90	70
46,42	1	20			4,8				150	125	100	75
34,6	0	7			0,6				140	125	88	80
27,09	0	12			8,5				160	90	85	
46,98	0	9			5,1				155	148	105	90
25,94	0	8			4,2							
34,42	0	8										
36,56	0	8			0,7				110	110	80	80
35,93	0	14			0,6				130	120	80	68
22,34	0	10			1,6				130	130	80	80
41,28	0	8			2,3				160	140	95	90
28,94	1	10			0,5				138	138	90	90
20,42	1	8			0,2				115	120	85	79
37,11	0	5	110	60	0,4	25,86	0	0	121	110	64	60
38,32	0	3	130	80	0		0	0	130	130	80	80
34,04	0	5	130	80	1,1	25,06	0	0	156	115	97	79
25,46	0	7	125	80	3,9	21,56	0	0	143	121	90	70
34,24	0	8	120	80	6		0	0	132	120	82	72
39,27	0	7			2,7	25,58	0	0	170	140	100	90
59,5	0	6	140	90	0,4		0	0	155	140	98	90
35,33	0	5					0	0				
37,69	0	8	130	90	2,7	26,45	0	0	148	128	98	80
33,35	0	4	140	86	2,4	20,02	0	0	140	138	86	80
65,31	0	11	140	86			0	0	140	140	86	86
52,77	1	7	110	60	3,1	24,87	0	0	132	110	88	60
42,46	1	8	108	82	0,4	24,76	0	0	115	108	82	70
38,54	0	8	120	80	3,9	24	0	0	146	110	80	60

41,25	1	7	120	85			0	0				
48,8	0	7	130	85	4	32,08	0	0	155	120	100	70
40,78	0	12	148	82	10		0	0	160	140	82	70
46,41	0	8	120	70	3,2	17,52	0	0	120	110	70	68
43,66	1	10	140	75	3,3	24,3	0	0	160	140	86	75
39,66	0	9	120	80	3,7	21,4	0	0	158	120	86	80
32,19	1	13			6,3		0	0	120	120	70	70
55,09	0	10	140	85	3,1	33,36	0	0	145	145	80	80
40,76	0	7	150	85	2		0	0	150	150	85	85
22,41	0	7			4,1	21,56	0	0				
42,75	0	6	130	90	5,5	21,36	0	0	150	130	90	80
26,86	1	4	130	85	0,3	21,29	0	0	120	120	80	80
29,08	1	11					0	0				
52,79	0	10	140	70	6,8	23,48	0	0	145	100	80	50
51,53	1	7	120	60	0,6	22,78	0	0	120	120	60	60
40,56	1	11	135	90	4,7	21,95	0	0	135	118	90	75
20,55	1	6	85	70	0	27,48	0	1	90	85	75	70
31,47	0	12	105	65	5,9	25,86	0	0	105	80	65	55
47,05	0	7			2,3		0	1	90	80	70	70
43,78	0	23			12,2		0	0	155	105	92	50
40,23	0	12	120	75	5,1	18,27	0	0	145	132	80	70
27,53	0	10	130	80	3,2	24,47	0	0	130	130	80	80
38,63	0	9	160	110	3,5	31,66	0	0	160	126	111	70
40,55	1	9	120	80	1,9		0	0	120	100	84	60
37,12	0	9	160	100			0	0	210	140	110	100
49,37	0	10	115	70	2,5	21,24	0	0	115	95	78	70
52,83	0	8			0,9		0	0	130	120	92	70
46,94	1	9	140	80	4,3	26,08	0	0	140	124	80	80
25,22	0	10	115	80	1,2		0	0	148	115	80	65
27,05	0	8	120	90	1,9	24,46	0	0	120	120	90	90
31,03	0	9	115	60	5	20,46	0	0	125	110	95	60
33,03	0	7	146	86	4	24,57	0	0	160	127	95	74
29,81	0	10	94	80	4,1	33,72	0	0	159	94	111	80

59,74	0	13	105	75	9,9	26,23	0	0	140	105	94	72
32,07	0	5	115	60	1,2	30,53	0	0	115	115	60	60
47,06	1	8	110	78	3,2	32,41	0	0	134	110	95	65
31,36	0	8	116	74	3	22,18	0	0	120	100	80	55
55,39	0	3	110	80	0	23,59	0	0	110	110	80	80
41,97	0	7	120	65	6,2	22,66	1	0	152	120	86	65
44,97	0	22	120	90	2,7	22,46	1	0	160	120	98	70
36,19	0	13	134	110	12	34,72	1	0	155	95	110	60
35,6	0	10	140	75	8,3		1	0	180	130	75	60
33,02	1	5			2,6				154	154	100	100
49,53	0	5			0,8				130	119	80	60
33,77	1	7			2,6				110	105	70	60
30	1	1										
57,92	0	20			4,9				144	110	100	60
39	0	7	120	70	6,8	20,24	0	0	120	113	76	70
31,33	0	10	140	70	5,3	27,6	0	0	173	135	88	69
36,92	0	10	120	80	4,9		0	0	166	113	101	74
27,17	0	9	125	80	4,2	20,93	0	0	130	116	71	84
29,71	1	11	115	90	5		0	0	130	115	90	80
30,03	1	6	110	76	1,7	21,9	0	0	132	102	93	63
49,79	1	6	122	80	0,9	32,44	0	0	122	117	75	75
28,82	0	12	60		10,55	27,4	1	1	153	60	95	69
67,01	1	30	180	72			1	1	119	68	71	39
63,74	1	9					0	0				
49,18	1	14	90	65	7,1	30,08	0	1	165	86	106	61
37,33	0	22	70	30	9,4	21,2	1	1	144	60	98	36

Crea max	HKR max	Leuk max	Tromb min	CRP max	Alb min	ABL	Rh
122	0,38	11,2	311	11		AB	neg
194	0,46	13,1	114	81		AB	neg
1183	0,39	14,5	18	34	32,9	AB	neg
727	0,39	8,6	145	56		AB	neg
143	0,42	6,8	61	78		AB	neg
93	0,35	4,9	76	50		AB	neg
484	0,43	7,8	56	109	29	AB	neg
390	0,44	8,1	93	72		AB	pos
138	0,41	9	67	81	25	AB	pos
945	0,45	11,3	27		25,9	AB	pos
373	0,5	12,6	83	97	29	AB	pos
206	0,39	8,9	112	48		AB	pos
152	0,56	19,8	26	115	24	AB	pos
356	0,41	15,5	99	280		AB	pos
320	0,44	6,3	101	51		AB	pos
117	0,5	10,6	55	42	34	AB	pos
329	0,39	5,3	114			AB	pos
71	0,42	5,6	52	115		AB	pos
108	0,41	7,1	41	83		AB	pos
444	0,35	8,2	102	35	26	AB	pos
350	0,41	12	95	25		AB	pos
297						AB	pos
151	0,4	8,7	47	114	30	O	neg
236	0,45	16,1	23	67		O	neg
452	0,41	7,2	76	45		O	neg
205	0,43	8,5	51	118		O	neg
102	0,45	20,9	9	66		O	neg
328	0,48	8,6	52	103		O	neg
235	0,54	17,9	35	134		O	neg
918	0,41	12	60	104		O	neg
929	0,44	15,5	45	92	25	O	neg
						O	neg

922	0,4	8,9	172	36	34	O	neg
866	0,35	13,1	67	30	30	O	neg
100	0,4	4,6	72	90		O	pos
756	0,56	14,8	17	203,6	19	O	pos
92	0,41	7,8	74	91		O	pos
						O	pos
538	0,62	23,3	11	111	22	O	pos
987	0,36	12,1	178	22	29	O	pos
208	0,45	8,9	35	26		O	pos
90	0,4	10,1	57	136	28	O	pos
111	0,4	11,3	26	55		O	pos
73	0,44	5	93	16		O	pos
730	0,43	9,1	332	29	31	O	pos
111	0,39	8,6	70	80		O	pos
121	0,45	4,8	80	41		O	pos
161	0,47	6,4	37	64		O	pos
125	0,45	5,1	152	81		O	pos
122	0,45	10,1	100	59		O	pos
90	0,37	4,1	99	103		O	pos
178	0,5	13,3	38	21	35	O	pos
91	0,42	4,5	54	68		O	pos
801	0,51	17,2	44	70	28,7	O	pos
80	0,36	5,4		39		O	pos
537	0,57	17,5	9	77		O	pos
392	0,55	14,8	17			O	pos
475	0,38	8,4	160	39		O	pos
139	0,52	7,8	64			O	pos
166	0,49	8,4	60			O	pos
830	0,57	23,5	144			O	pos
165	0,61	11,3	19	49		O	pos
1241	0,26	9,4	33	45		O	pos
227	0,38	9,1	134	61		O	pos
1290	0,4	12,9	68	133		O	pos

265	0,41	10,1	85	36	34	O	pos
94	0,39	6,9	88	97	31	O	pos
134	0,53	44,7	24	95	28	O	pos
102	0,46	9,4	63	87		O	pos
108			154	38		O	pos
70	0,42	7,9		35		O	pos
119	0,4	5,3	88	53		O	pos
478	0,36	8	127	23		O	pos
138	0,38	6,3	36	123		O	pos
198	0,41	5,8	70	68	28	O	pos
421	0,44	9	167	110		O	pos
792	0,34	13,8	30	42		O	pos
116	0,38	5,3	103	118		O	pos
109	0,42	6,4	71	43		O	pos
100	0,38	5,9	84	29		O	pos
621	0,37	10,4	154	56		O	pos
222	0,43	8,3	39	56		O	pos
224	0,39	13,9	239	20	39,7	O	pos
72	0,46	10,5	95	48		O	pos
147	0,46	3,8	81	37		O	pos
487	0,42	8,4	97	45		O	pos
200	0,41	8,2	36	86		O	pos
223	0,42	8,5	75	145	28	O	pos
206	0,43	5,4	54	80	37,2	O	pos
102	0,39	9,9	63	43		O	pos
515	0,54	43,3	10	75	25	O	pos
128	0,45	6,9	24	113		O	pos
581	0,33	7,5	228	51	35,8	O	pos
85	0,39	7,5	36	87	26	O	pos
1156	0,56	21	19	109	24	O	pos
1246	0,4	12,7	42	68		O	pos
1071	0,39	18,3	28	33		O	pos
264	0,5	7,1	51	62,3		O	pos

292	0,45	6,9	181	28	43	O	pos
126	0,49	17,6	27	27	28	O	pos
109						O	pos
88	0,4	6,8	31	112		O	pos
568	0,52	15,5	54	81	27	O	pos
90	0,43	4,9	87	12		O	pos
75	0,38	6,5	80	100		O	pos
237	0,46	9,9	52	76		O	pos
150	0,49	16,2	19	75		O	pos
139	0,42	10,2	66	95	37	O	pos
260	0,45	11,5	56	45	30	O	pos
123	0,47	6,5	80	39	27	O	pos
71	0,42	6,2	35	73		O	pos
122	0,41	10,8	75	64		O	pos
1281	0,47	8,2	47	169		O	pos
241	0,43	26,8	13	214		O	pos
648	0,4	19,7	18	43		O	pos
267	0,47	9,8	78	69	36	B	neg
496	0,39	10,3	96	46		B	neg
296	0,43	14,1	60	85	30	B	neg
621	0,59	16,6	15	52	28	B	neg
95	0,35	5,9	67	111	30	B	neg
749	0,43	14,7	59	34	25	B	neg
173	0,45	13,3	69	107		B	neg
197	0,42	8,9	100	63	34	B	pos
529	0,47	17,9	55	29	24	B	pos
489	0,41	16,7	52	66		B	pos
67	0,36	6,8	60	109		B	pos
353	0,51	10,1	97	95	31	B	pos
1304	0,41	19,4	71	47	22	B	pos
182		9,2	141	45		B	pos
310	0,44	10,3	168	96		B	pos
502	0,41	11,8	264	58		B	pos

500	0,41	10,7	122	44		B	pos
72	0,4	4,4	64	37	28	B	pos
590	0,39	8,7	44	118	23	B	pos
73	0,43	9,3	135	116		B	pos
79	0,38	8,6	136	126	34	B	pos
515	0,64	37,7	20	27	29	B	pos
135	0,44	8,4	61	136	35	B	pos
332	0,51	26,1	20	80		B	pos
123	0,43	5,7	44	137		B	pos
156	0,51	9,3	61	85		B	pos
138	0,41	7,9	84	156		B	pos
218	0,32	8,3	160	50		B	pos
218	0,45	12,1	213	104		B	pos
184	0,45	6,9	82	32	36,8	B	pos
170	0,57	11	70	21		B	pos
292	0,38	7,7	142	20	33,5	B	pos
539	0,42	8,8	108	71	35	B	pos
324	0,39	5,9	230	54		B	pos
393	0,44	7,5	102		34	B	pos
77						B	pos
541	0,5	12,1	13	120	25	B	pos
1105	0,39	22,9	10	35		B	pos
1537		50,3	18			B	pos
171	0,37	8,5	66	13		B	pos
361	0,41	9,2	42	65	23	B	pos
356	0,42	8,3	28	83	27	B	pos
78	0,45	7,7	3	69	39	B	pos
91	0,41	6,8	57	90	39	B	pos
543	0,41	13,1	33	120		B	pos
473	0,56	17,4	24	101	26	B	pos
333	0,4	9,7	98	67		B	pos
1042	0,54	35,4	11	113	27	B	pos
544	0,43	12,3	192	26	32,8	A	neg

237	0,42	8,4	205	36		A	neg
148	0,45	12,9	163	13		A	neg
281		8,6	89	22		A	neg
105	0,4	7,5	93	31		A	neg
181	0,46	11,2	46	32		A	neg
290	0,41	7,3	98	92		A	neg
126	0,44	10,1	56	84		A	neg
405	0,51	14,5	39	115	25,6	A	neg
943	0,42	13,5	74	92		A	neg
493	0,41	10,7	121	19		A	neg
252	0,38	7,1	348	33		A	neg
120	0,38	10,4	94	175	30	A	neg
1026	0,62	32,4	15			A	neg
254	0,43	11,1	39	101		A	pos
178	0,58	18,1	40	100	26	A	pos
222	0,44	17,1	10	118	22	A	pos
241	0,39	9,7	109	100	31,8	A	pos
94	0,43	7,2	68	130	32	A	pos
792	0,39	13,2	29	195	21	A	pos
81	0,42	9,6	73	120	29	A	pos
121	0,45	9,8	128	160		A	pos
93	0,41	9,1	96	124		A	pos
265	0,37	9,8	68	36		A	pos
85	0,43	8,4	82	68		A	pos
233	0,42	7	56	131		A	pos
363	0,42	10,2	182	40		A	pos
127	0,38	10,9	95	77		A	pos
155	0,41	8	270	139		A	pos
506						A	pos
293	0,41	19,7	9	59		A	pos
102	0,46	6,7	106	32		A	pos
164	0,54	11,4	30	11		A	pos
466	0,42	10,4	150	33	30	A	pos

398	0,38	14,7	71	63	27	A	pos
126	0,39	7,8	72	143		A	pos
441	0,38	11,2	55	50	28,9	A	pos
138	0,41	13,4	165	42		A	pos
509	0,43	9,1	68	93	37,4	A	pos
403	0,64	17,6	52	19	29	A	pos
231	0,49	19,4	26	125		A	pos
397	0,47	15,8	282	120	31	A	pos
393	0,46	11,4	137	23		A	pos
607	0,43	17	25	103		A	pos
630	0,38	9,3	192			A	pos
171	0,51	14,5	38	52		A	pos
142	0,38	10,9	98			A	pos
396		11,3	109	40		A	pos
200	0,46		42	89		A	pos
350	0,42	7,4	21	58		A	pos
134	0,44	7,4	201	27		A	pos
113	0,42	7,8	64			A	pos
340	0,41	8,3	57	27	41,5	A	pos
275	0,38	11,6	29	200	27	A	pos
98	0,48	8,9	118	70	45	A	pos
74	0,41	8,9	105	82	34	A	pos
149	0,5	8,3	55	55		A	pos
756	0,43	9,8	84	82	33	A	pos
290	0,41	7,7	51	61		A	pos
212	0,45	8,4	70	42		A	pos
163	0,43	6	73	83		A	pos
319	0,46	11,4	109	103	30	A	pos
124	0,39	9,8	80	84		A	pos
182	0,42	13,3	53	133		A	pos
223	0,4	9,1	67	42	30	A	pos
274	0,46	10,3	74	103	27	A	pos
915	0,35	11,6	112	88		A	pos

93	0,44	4,5	46	124		A	pos
376	0,42	11,4	111	83	34	A	pos
972	0,34	13	85	22	27	A	pos
80	0,39	6,2	36	55		A	pos
238	0,41	11,1		32		A	pos
181	0,46	9	69	57		A	pos
379	0,41	10,1	63	86		A	pos
124	0,46	8	89	69	35,5	A	pos
83	0,42	11,5	89	55	33	A	pos
349	0,39	7,7	167	32		A	pos
1645	0,43	16,1	54	32		A	pos
90	0,42	9,6	70	53	31,4	A	pos
369	0,37	11,8	70	108		A	pos
484	0,43	12,8	378			A	pos
71	0,43	6,2	74	111		A	pos
515	0,46	7,4	45	75	24	A	pos
119	0,42	8,6	130	70		A	pos
559	0,5	15	56	68	30,9	A	pos
219	0,51	12,1	78	68	29	A	pos
1011	0,62	29,5	10	109	19	A	pos
1168	0,37	10,1	65	31		A	pos
380	0,44	10,4	106	17		A	pos
119	0,35	9,5	139	49		A	pos
264	0,34	6,8	96	96		A	pos
219	0,46	12,1	122	77		A	pos
238	0,43	8	38	44		A	pos
152	0,46	9,2	147	77		A	pos
720	0,37	10,5	314	53		A	pos
285	0,42	8,2	111		39	A	pos
114	0,5	9,2	62	64		A	pos
369	0,49	13,3	30	15		A	pos
484	0,37	5,8	36	98	26	A	pos
455	0,6	22,6	18	56	29	A	pos

725	0,47	20,4	17	72	25	A	pos
98	0,45	7	104	104	32	A	pos
458	0,42	11,2	139	53	32	A	pos
259	0,5	8,2	36	87		A	pos
92	0,4	6	108	94		A	pos
725	0,44	15,6	71	62		A	pos
1443	0,5	19	12	181	26	A	pos
1285	0,49	23,2	9	37	27	A	pos
1324	0,4	9,6	108	75	30,4	A	pos
185	0,46	11,7	26	96		A	pos
112	0,41	9,5	107	68	38	A	pos
197	0,48	13,5	66	39	29	A	pos
75						A	pos
350	0,57		36	154		A	pos
524	0,39	12,9	24	26	24	A	pos
512	0,41	11,5	86	75		A	pos
645	0,47	10,8	86	45		A	pos
704	0,37	14,6	99	182	33	A	pos
426	0,58	19,8	77	108		A	pos
175	0,4	8,6	74	29	30	A	pos
89	0,4	7,2	75	93	28	A	pos
641	0,66	39,1	4	44,8	24	A	pos
265	0,59	35,5	37	82,4	21	A	pos
155			53			A	pos
950	0,54	8,1	22	194	22	A	pos
463	0,53	38,6	25	69,7	11	A	pos
